# Validation of the Persian version of family health climate scale (FHC-Scale) in Iranian families

**DOI:** 10.1186/s12889-020-09931-8

**Published:** 2020-12-03

**Authors:** Akram Kharazmi, Jeannine M. Brant, Moosa Sajjadi, Mahdi Moshki, Leila Sadegh Moghadam

**Affiliations:** 1 Social Development and Health Promotion Research Center, Gonabad University of Medical Sciences, Gonabad, Iran; 2 Montana State University, College of Nursing, Bozeman, MT USA; 3 Department of Medical-Surgical Nursing, Faculty of Nursing, Social Development & Health Promotion Research Center, Gonabad University of Medical Sciences, Gonabad, Iran; 4 Department of Health Education and Health Promotion, School of Health, Social Development and Health Promotion Research Center, Gonabad University of Medical Sciences, Gonabad, Iran; 5 Department of Gerontology,School of Nursing, Social Development and Health Promotion Research Center, Gonabad University of Medical Sciences, Gonabad, Iran

**Keywords:** Validation, Psychometric, Family health climate scale, FHC-scale, Iran

## Abstract

**Background:**

Family health is an important issue which has attracted researchers from different fields. The present study aimed to validate the Persian version of the Family Health Climate Scale (FHC-Scale).

**Methods:**

In this methodological research, a total of 620 individuals presenting to Comprehensive Healthcare centers affiliated with Mashhad University of Medical Sciences and Gonabad University of Medical Sciences were selected through random multistage sampling. Validation of the FHC-Scale was performed. First, the original scale was translated and back-translated. Then its content validity and construct validity were assessed using exploratory and confirmatory factor analysis. Reliability was assessed using internal consistency and stability. Data were analyzed using SPSS version 20 (IBM Corp., Armonk, NY, USA) and LISREL version 8.5 (SSI Inc., Skokie, IL, USA).

**Results:**

Results of exploratory factor analysis showed that “physical activity” of family health climate scale (FHC-PA) has three dimensions: value, cohesion and information explaining 61.99% of the variance. “Nutrition” of family health climate scale (FHC-NU) had four dimensions of value, communication, cohesion and consensus explaining 66.19% of the variance. Internal consistency of the dimensions of (FHC-PA) ranged 0.82–0.85 and that for FHC-NU ranged 0.82–0.84. Confirmatory factor analysis revealed goodness of fit and confirmed family health climate scale (Nutrition and physical activity).

**Conclusion:**

Results of the study revealed that the FHC-Scale has appropriate reliability and validity for Iranian families. Therefore, the Persian version of the scale can be used for assessing health-related aspects of family.

**Supplementary Information:**

The online version contains supplementary material available at 10.1186/s12889-020-09931-8.

## Background

The family is a complicated emotional system that is considered the major pillar and the initial organization of any society, where human emotions and intimate relations emerge. It can be said that family functioning affects society functioning in a direct and major way. In other words, a healthy society is composed of healthy families. Members of a family are connected through biological, legal, emotional, geographical and historical bonds. As compared with other social groups, entering a family in the traditional sense is possible through giving birth to a child, adopting a child, caring for a child or getting married [1]. In Iran, the family is an indicator of the cultural value system. Recent decades have been exposed to structural changes that have affected all aspects of the family including communication among members, the emotional importance of members for one another, the meaning of family, economical functioning of family and the sexual roles [2]. Christensen believes the family is the cultural reflection of thousands of years of efforts to develop the members, improve their mental health, and reduce high-risk behaviors [3]. Therefore, given the continuous interactions that family members have within the family and with the society, it appears that family is the main focus of health in the society.

Based on a model developed in Iran, a healthy family is one with positive characteristics such as using healthy parenting styles, having skills for consulting with one another and decision making, and ensuring physical, mental and emotional needs of the members [4]. Healthy family functioning helps children to acquire a positive identity, increase independence, and reduce the incidence of behavioral and communication problems [5]. Currently, the comprehensive definition of family health is as follows: family health comprises a family’s quality of life, health of each member, family interactions, spiritual indicators, nutrition, overcoming problems, living environment, entertainment, daily activities, sleep, and sexual desires [6].

Studies show that healthy functions of the family directly affect needs, objectives, satisfaction with life and emotional relations [7], and affect factors such as structure, roles, communication, perception and dynamic personality of members [8]. Research on the family has mainly focused on the influence of family members on one another, especially between couples and parent-child relations [9, 10].

Assessing family health is one of the major objectives of family therapists, that is used to identify challenges and propose solutions to help families reach their specific goals. A variety of tools are used to assess function and health of families, but no consensus has been reached on a single tool for this purpose [11]. One of the most important and influential factors for family health is maintaining physical activity and healthful nutrition [12, 13], but studies overall show that these topics are not well addressed in families [14]. Studies show Iranian families also pay less attention to physical activity and nutrition [15].

One assessment tool, which fits within the operational context of the family is the family health climate scale (FHC-Scale), developed by Niermann et al. (2014) [16]. The FHC-Scale, developed in Germany, was based on Bandura’s social cognitive theory, which assumes that the environment and the individual determine their mutual relationship and affect one another’s healthy behavior. The FHC-Scale has two scales, physical activity (FHC-PA) (14 items) and nutrition (FHC-NU) (16 items) scored on a Likert scale from totally false (0) to totally true (3).

The FHC-PA scale has three dimensions: 1)“Value” reflects the importance of physical activity for all members of the family, 2) “Cohesion” covers common physical activities and leisure activities of family members, and 3) “Information” refers to the amount of searching for, sharing and using information related to sports and physical exercises. The FHC-NU scale has four dimensions: 1) “Value” focuses on family’s emphasis on improving nutrition in their daily diet, 2) “Cohesion” reflects regular eating sessions of the family and the importance of eating together, 3) “Communication” covers the support and encouragement of family members for having a balanced diet 4) “Consensus” comprises the acceptance of family members’ interests in nutrition-related behaviors [16].

As mentioned, family health is the foundation of individual health within a given society, and measuring family health is an important area of research. The FHC-Scale is an attractive tool to measure family health in that it is comprehensive but has fewer items, which makes it more feasible for use, and reliability and validity indices are also established. Therefore, the purpose of this study was to translate and validate the Persian version of FHC-Scale in Iranian families.

## Methods

### Participant recruitment

The study population was comprised of families living in Mashhad and Gonabad cities in Iran. Given that Mashhad is the capital of Khorasan Razavi Province and people of different ethnicities live there, the results of the study can potentially be better generalized across the Iranian population. Several healthcare centers were first selected randomly from Mashhad and Gonabad, and then participants were selected by convenience and purposive sampling from among families covered by these centers. Sampling continued until sample size was appropriate for confirmatory factor analysis. The inclusion criteria were being Iranian, having at least one child over 14 years. Families with a history of divorce or history of admission to a psychiatry center were excluded. Accordingly, 620 families were finally selected. One member (father, mother, or child) from each family participated in the study. The present study was approved by the Gonabad University of medical science Research Ethics Committee (code: IR.GMU.REC.1395.102). Informed written consent was obtained from all participants.

### Translation of the scale

The present study used Wilde et al.’s model (2005) [17] for translating and validating the scale. After obtaining the written approval of the scale developer, the FHC-Scale (FHC-PA and FHC-NU) was translated to Persian. Two translators who were competent in both German and Persian translated the scale to Persian. The two translations were compared and contrasted, and then merged into one final translation. In the next step, the Persian version was translated to German by two competent translators who had no contact with the first translators. The two translations were compared with the original scale by a lead translator and confirmed. Finally, psychometric assessment of the translated scale was conducted using face validity, content validity, construct validity (exploratory and confirmatory factor analyses), internal consistency and stability.

### Content validity

The translated scale was next given to 10 faculty members experienced in scale development and familiar with family health to assess content validity of the items. They expressed their opinion of the relevance of each item based on Waltz and Bussel (1983) content validity index, so that content validity of the scale could be measured [18]. They also assessed and confirmed face validity of the scale. Following the review by experts, the scale was given to 10 family members for feedback on simplicity of the scale and understandability of items so that possible ambiguities could be further refined. The Persian version of FHC-Scale was finalized at this stage without major changes in its items.

### Data collection and analysis

Participants were selected and briefed about the study objectives, and written informed consent was obtained. A demographics questionnaire (age, place of residence, economic status, parents’ occupation, education level, family size, and level of daily activity) and the Persian version of the FHC-Scale was administered to one of the three family members (father, mother, or child) in every family. Completion of the questionnaire took 15 to 20 min. Internal consistency reliability was measured using Cronbach’s alpha, and stability was measured using an interclass correlation coefficient (ICC) with a two-week interval between measures. The literature notes that two or 3 weeks is an appropriate interval between the two tests [19].

Construct validity of the FHC-Scale was assessed and confirmed using exploratory (EFA) and confirmatory factor analyses (CFA). To this end, the study population was divided into two nearly equal subsamples: 300 individuals were assigned to calibration sample and 320 individuals were assigned to validation sample. EFA was used to analyze the calibration sample so that the model could be to examined and modified as necessary. The validation sample was used for validating the fit model.

Construct validity was performed with confirmatory factor analysis in Lisrel® version 8.5. Maximum likelihood estimation was used to assess model fit, using a variety of fitness indices as recommended in the literature [20, 21]. The most important fitness indices used included Chi-square, SRMR, NFI, CFI, AGFI, GFI, and RMSEA. Appropriate values should be less than 0.06 for RMSEA, less than 0.08 for SRMR, and 0.9 or ideally greater than 0.95 for other indices [21, 22]. Before conducting CFA the assumptions of maximum likelihood estimation were assessed. Item distributions were inspected for multivariate normality using p-p plot and indicated normal distribution of the data. The correlation between the items for both scales were less than .80.

## Results

The sample included 620 family members. Mean age of the parents was 43.5 (10.8) and for the children 21.3 (6.5) years. The majority of fathers did not have a diploma, whereas the mothers were better educated noted by having a diploma and/or college degree. Mothers comprised 33.9% of the sample; fathers 26.9% and children 39.2%. The demographic details of the study subjects are presented in Table 1.

**Table 1:** Demographic characteristics of the participants (*n* = 620)

Variable	Mean	SD (Range)
Age(year)	34.8	14.3 (14–85)
Duration of the family formation (year)	21.9	10.24 (1–68)
Physical activity per day(Hour)	1.1	1.1 (0–8.0)
Category	**N**	**%**
City		
Mashhad	373	60.2
Gonabad	247	39.8
Family income		
Less than enough	65	10.5
Enough	485	78.2
More than enough	67	10.8
Education level (for father)		
Illiterate	66	10.8
Under diploma	190	31
Diploma	179	29.2
Bachelor’s degree	147	24.0
Master’s degree.	21	3.4
PhD degree	9	1.5
Education level (for mother)		
Illiterate	61	9.9
Under diploma	191	31.0
Diploma	226	36.7
Bachelor’s degree	126	20.5
Master’s degree.	12	1.9
PhD degree	0	0
Occupation (for father)		
Self-employed	299	48.9
Employee	141	23.0
Worker	70	11.4
Retired	73	11.9
Unemployed	13	2.1
Other	16	2.6
Occupation (for mother)		
Self-employed	29	4.7
Employee	73	11.8
Worker	6	1.0
Retired	45	7.3
Housewife	457	73.8
Other	9	1.5
Role in family		
Father	167	26.9
Mother	210	33.9
Daughter	140	22.6
Son	103	16.6
Meals Together		
breakfast	2	0.3
Lunch	40	6.5
dinner	141	22.7
Two meals	103	16.6
All three meals	278	44.8
None of meals	55	8.9

### Scale validity and reliability

Based on the scale reviews of the 10 experts regarding item relevance, the content validity index was found at 0.807 for the FHC-PA and 0.893 for the FHC-NU, indicating favorable content validity.

Table 2 illustrates the mean values of the physical activity and nutrition subscales and their dimensions in Iranian families as well as assessment of their internal consistency reliability as measured by Cronbach’s alpha. All scales performed at 0.82 or greater. We also provided the inter-item correlation matrix for both the FHC-PA and the FHC-NU scales (See Additional file 1).

**Table 2:** Mean scores and values of Cronbach’s alpha, composite reliability and average variance extracted in the FHC-NU and FHC-PA scales in Iranian families (*n* = 620)

scale	Dimension	Mean (SD)	α	AVE	CR
FHC-NU scale	**Value**	9.04 (2.51)	0.84	0.55	0.83
	**Cohesion**	9.57 (3.39)	0.82	0.51	0.83
	**Communication**	9.22 (2.46)	0.83	0.58	0.84
	**Consensus**	5.75 (2.18)	0.83	0.54	0.79
FHC-PA scale	**Value**	9.24 (3.30)	0.83	0.51	0.81
	**Cohesion**	11.10 (3.06)	0.85	0.55	0.75
	**Information**	5.95 (3.15)	0.82	0.57	0.74

*SD* standard deviation, *α* Cronbach’s alpha, *AVE* average variance extracted, *CR* composite reliability

The traditional indicator for the reliability is Cronbach’s alpha, but the composite reliability (CR) is more adequate to the Lisrel program. Because it prioritizes the variables according to their reliabilities, while the Cronbach’s alpha is more sensitive to the number of variables in each construct. The CR value of 0.7 or greater considered as good reliability [10].

Convergent validity was assessed by Average Variance Extracted (AVE) the mean variance is extracted and measures how much manifest variables correlate positively with their respective latent variables (mean of the correlation). The literature considers there is convergent validity when the AVE value is greater or equal to 0.50 [10]. All dimension of the both scales have proper AVE and CR values (Table 2).

Stability reliability of the FHC-scale was assessed by a test-retest methodology with 30 individuals. The ICC was 0.89 for the FHC-PA and 0.94 for the FHC-NU, indicating favorable stability for these scales.

### Factor analysis

Exploratory factor analyses were performed with SPSS 20 using the Principal Component method with Varimax rotation. The Kaiser-criterion (eigenvalue > 1) yielded three factors for the FHC-PA Scale and four factors for the FHC-NU Scale with eigenvalues greater than one respectively. Separate analyses were carried out for the physical activity scale and the nutrition scale. Exploratory factor analysis of the calibration sample (*n* = 300) showed that like the original scale, the FHC-PA is composed of three dimensions (value, cohesion, and information) and the FHC-NU is made-up of four dimensions (value, cohesion, communication, and consensus). Compared to the original scale, items of each dimension identically matched (Table 3). The three dimensions of FHC-PA scale explained 61.99% of the variance, and four dimensions of FHC-NU scale explained 66.19% of the variance. For both scales, the requirements for performing EFA were fulfilled (FHC-PA: KMO = 0.853, Bartlett’s test of sphericity X^2^ = 1763.13, *P* < 0.001; FHC-NU: KMO = 0.900, Bartlett’s test of sphericity X^2^ = 2235.75, *P* < 0.001).

**Table 3:** Factors, Items, and Factor Loadings in FHC-PA and FHC-NU scales

Scales	Factors	Items	Mean (SD)	Factor Loading
**FHC-PA**	Value	we make a point of being physically active during daily life.	1.70 (.91)	0.770
		it is normal to be physically active on a regular basis.	1.96 (.83)	0.754
		it goes without saying that we exercise and are physically active on a regular basis.	1.75 (.88)	0.772
		it is normal to be physically active in our leisure time.	1.88 (.81)	0.703
		we agree that physical activities are part of daily life.	1.94 (.78)	0.569
	Cohesion	we like being together during physical activities (e.g. bike tours, hikes).	2.26 (.76)	0.676
		we enjoy exercising together.	2.19 (.78)	0.823
		we have fun doing physical activities together (e.g. bike tours, hikes).	2.17 (.81)	0.834
		we find it very pleasant to be physically active together.	2.24 (.75)	0.797
		we like spending time together in sports activities.	2.17 (.75)	0.661
	Information	we watch TV-programs on physical activity and exercise.	1.67 (.96)	0.715
		we explicitly look for the latest information on physical activity and exercise to stay up to date.	1.43 (.95)	0.851
		we collect information (e.g. on the internet) on physical activity and exercise.	1.41 (.93)	0.790
		we read newspaper or magazine articles on fitness, physical activity, and exercise.	1.58 (.98)	0.723
**FHC-NU**	value	a healthy diet plays an important role in our lives.	2.33 (.83)	0.780
		we naturally pay attention to eating healthfully.	2.32 (.76)	0.764
		we routinely eat healthfully.	2.22 (.78)	0.741
		it is normal to choose healthful foods.	2.21 (.76)	0.723
	Communication	we are interested in articles (e.g. in magazines) on healthful nutrition.	1.77 (.95)	0.599
		we remind each other to pay attention to a healthful diet.	1.93 (.87)	0.694
		we talk about which foods are healthful.	2.00 (.86)	0.779
		we support each other to refrain from unhealthful things.	1.99 (.95)	0.672
		we talk about how to eat healthfully.	1.99 (.84)	0.803
	Cohesion	we appreciate spending time together during meals.	2.25 (.76)	0.752
		everybody enjoys having meals together.	2.38 (.68)	0.832
		eating together is a part of our daily family life.	2.22 (.81)	0.743
		we enjoy meals most when we sit at the same table.	2.35 (.72)	0.709
	Consensus	we rarely argue about food- or diet-related matters.	1.87 (.91)	0.584
		we agree on diet and nutrition.	1.98 (.86)	0.635
		we usually agree on meals and food choices.	2.03 (.87)	0.574

**Fig. 1:**
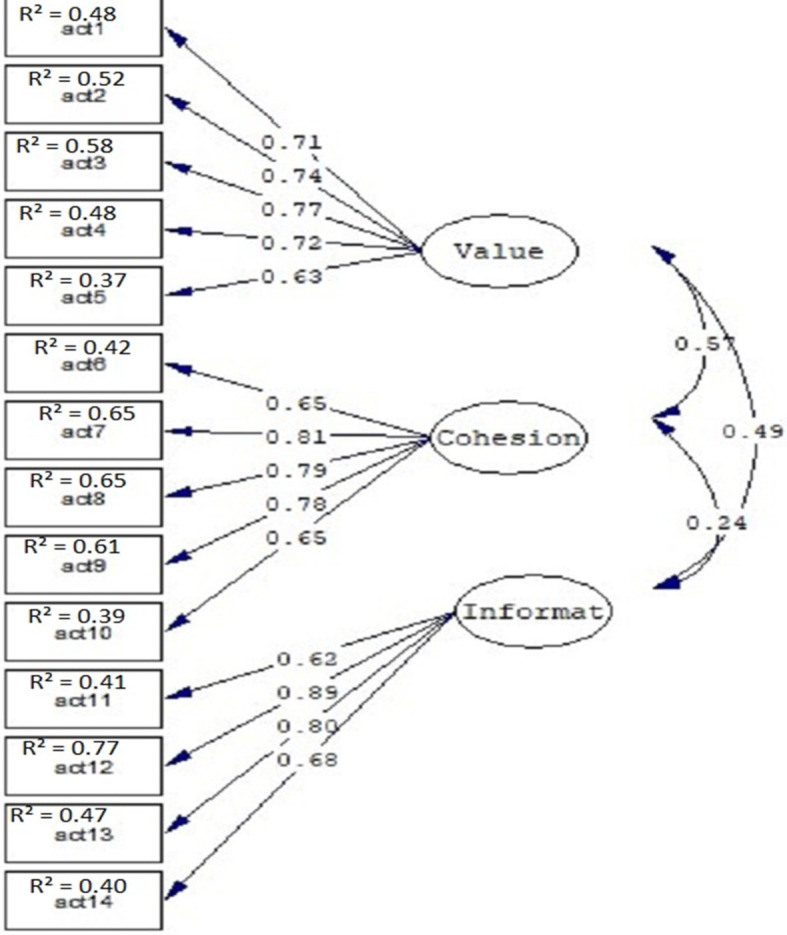
FHC-PA – standardized factor loadings and interfactor correlations

**Fig. 2:**
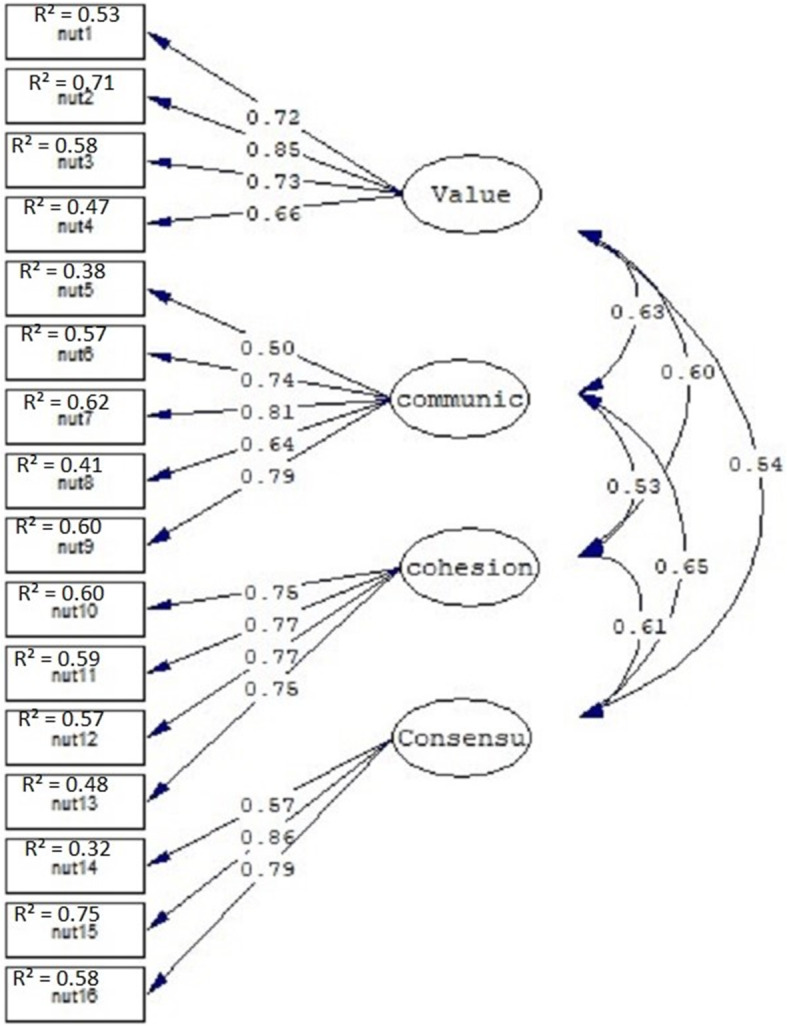
FHC-NU – standardized factor loadings and interfactor correlations

To assess construct validity of the scale, CFA was performed using Lisrel-8.5. According to the factor analysis performed on the calibration sample, no change was made in items or in their placement as factor loading for all items was > 0.5 and fit indices for both scales were highly favorable. CFA was then performed on the validation sample (*n* = 320) for further confirmation and validation. The fitness indices showed goodness of fit for the scales. The confirmatory factor analysis results for FHC-PA and FHC-NU scales are presented in Tables 4 and 5. Based on the fitness indices found, the FHC scale has a highly favorable validity, and is approved for use in Iranian society. The models with factor loadings and factor correlations are shown in Figs. 1 and 2.

**Table 4:** Confirmatory Factor analysis Fit indices for the FHC-PA scale in Iranian families

Index sample	(Χ2/df), *p*	RMSEA (90%CI)	CFI	GFI	AGFI	NFI	SRMR
FHC-PA scale (calibration sample *n* = 300)	(2.72), *p* < 0.001	0.074 (0.06–0.08)	0.96	0.91	0.88	0.94	0.057
FHC-PA scale (validation sample *n* = 320)	(2.63), *p* < 0.001	0.074 (0.06–0.08)	0.97	0.92	0.88	0.95	0.058

**Table 5:** Confirmatory Factor analysis Fit indices for the FHC-NU scale in Iranian families

Index sample	(Χ2/df), *p*	RMSEA (90%CI)	CFI	GFI	AGFI	NFI	SRMR
FHC-NU scale (calibration sample *n* = 300)	(2.10), *p* < 0.001	0.059 (0.05–0.07)	0.98	0.92	0.89	0.96	0.050
FHC-NU scale (validation sample *n* = 320)	(1.85), *p* < 0.001	0.050 (0.04–0.06)	0.98	0.92	0.91	0.97	0.042

## Discussion

Family health is one of the most important issues in any society, and its study is of considerable importance. However, despite its importance, family health research has not attracted much attention in Iran, which can be due to the lack of suitable and practicable scales to measure family health. The present study was conducted to translate and validate the Persian version of the Family health climate scale in Iranian society. When a scale is translated or is to be used in another culture or society, it must be validated [23, 24].

The present study results showed that the Persian version of FHC scale (FHC-PA and FHC-NU) and its dimensions have favorable content validity, construct validity and reliability for use in Iranian society, and like the original scale, is approved [16].

For content validity of the scale, CVI was found 0.807 for FHC-PA and 0.893 for FHC-NU, which exceed 0.79 and indicate favorable CVI values [19]. The Farsi version of FHC scale also had favorable Cronbach’s alpha coefficients (0.86 and 0.90 respectively), which exceeded the 0.7 limit and coefficients higher than 0.8 suggest that the scale has a high internal consistency [19].

Regarding construct validity, the exploratory factor analysis results showed that the translated scale totally matches the original scale in terms of number of dimensions and items within each, and elimination or replacement of the items was not necessary. This shows that the original scale was properly developed and can be used in Iranian society.

The CFA results showed that the original FHC model is also totally appropriate for use in Iranian families, and fitness indices obtained indicated total fitness of this scale and its dimensions. When conducting CFA, Lisrel usually provides recommendations for modification and improvement of the model that can be useful. However, given the model fit in the present study, change or replacement of items was not necessary.

The fact that a scale is translated and validated in another society has a favorable fit and has no need for change has also been found in other studies [23, 25], which agrees with the present study results. The concepts of nutrition and physical activity that are assessed by the FHC scale appear not to be widely different and can be used in different societies.

While the versions of the FHC-Scale seem to be consistent across languages and culture, other scales have not translated as easily. For example, in a study by Sajjadi et al. (2014) on translation and validation of the Uncertainty of Illness Scale in patients with cancer, some items have to be eliminated for use in Iranian society [26]. In a study conducted by Klemenc-Ketis et al. (2018) to validate a translated scale into Slovenian language, the initial model did not have a goodness of fit, but fitness indices became favorable with different dimensions in the translated version from the original scale [27]. These variable findings suggest how different a translated scale is from the original depends on several factors such as the concept studied, methodology and accuracy of developing the original tool, use of language, cultural differences, and other factors.

### Limitations

One of the study limitations was sampling in one province only, which may affect generalizability of the results to the whole country. Another limitation was the self-reporting questionnaire, where some participants may not have accurately answered the questions.

## Conclusion

The present study results showed that the FHC scale (including FHC-PA and FHC-NU scales) has favorable validity and reliability for use in Iranian society, and since it has relatively few items, it is easy for participants to complete and can be used to assess the health status of Iranian families.

## Supplementary Information

**Additional file 1.** Inter-items correlation matrix. This file included two tables that shows inter-items correlation matrix for both FHC-PA and FHC-NU scales.

## Data Availability

The datasets used and/or analyzed during the current study are available from the corresponding author on reasonable request.

## Abbreviations

FHC: Family health climate
FHC-PA: Family health climate – Physical activity
FHC-NU: Family health climate- Nutrition
SRMR: Standardized root-mean-square residual
NFI: Normed Fit Index
CFI: Comparative fit index
AGFI: Adjusted goodness of fit index
GFI: Goodness of fit index
RMSEA: Root-mean-square error of approximation
CVI: Content validity index
ICC: Interclass Correlation Coefficient
