# Supplementary material for: Validation of the Persian version of family health climate scale (FHC-Scale) in Iranian families
Source: BMC Public Health. 2020 Dec 3;20:1854. doi: 10.1186/s12889-020-09931-8 (PMC7713167; doi:10.1186/s12889-020-09931-8)
Supplement: Supplementary file 1 — Additional file 1. Inter-items correlation matrix. This file included two tables that shows inter-items correlation matrix for both FHC-PA and FHC-NU scales. [file 12889_2020_9931_MOESM1_ESM.docx]

| Items | NU1 | NU2 | NU3 | NU4 | NU5 | NU6 | NU7 | NU8 | NU9 | NU10 | NU11 | NU12 | NU13 | NU14 | NU15 | NU16 |
| --- | --- | --- | --- | --- | --- | --- | --- | --- | --- | --- | --- | --- | --- | --- | --- | --- |
| NU1 | 1.000 | .635 | .519 | .453 | .217 | .323 | .322 | .262 | .334 | .333 | .361 | .353 | .339 | .219 | .337 | .294 |
| NU2 |  | 1.000 | .617 | .546 | .284 | .422 | .407 | .346 | .392 | .382 | .369 | .425 | .413 | .323 | .412 | .366 |
| NU3 |  |  | 1.000 | .503 | .295 | .380 | .376 | .303 | .400 | .299 | .269 | .279 | .312 | .280 | .305 | .294 |
| NU4 |  |  |  | 1.000 | .336 | .425 | .350 | .245 | .345 | .343 | .332 | .325 | .333 | .264 | .277 | .248 |
| NU5 |  |  |  |  | 1.000 | .439 | .380 | .286 | .366 | .266 | .137 | .175 | .143 | .187 | .315 | .296 |
| NU6 |  |  |  |  |  | 1.000 | .611 | .458 | .537 | .352 | .280 | .383 | .348 | .319 | .415 | .395 |
| NU7 |  |  |  |  |  |  | 1.000 | .507 | .662 | .306 | .274 | .298 | .317 | .338 | .424 | .380 |
| NU8 |  |  |  |  |  |  |  | 1.000 | .554 | .316 | .231 | .277 | .306 | .324 | .343 | .280 |
| NU9 |  |  |  |  |  |  |  |  | 1.000 | .378 | .301 | .296 | .278 | .351 | .454 | .409 |
| NU10 |  |  |  |  |  |  |  |  |  | 1.000 | .636 | .572 | .495 | .257 | .421 | .396 |
| NU11 |  |  |  |  |  |  |  |  |  |  | 1.000 | .569 | .586 | .280 | .332 | .333 |
| NU12 |  |  |  |  |  |  |  |  |  |  |  | 1.000 | .611 | .249 | .399 | .404 |
| NU13 |  |  |  |  |  |  |  |  |  |  |  |  | 1.000 | .327 | .416 | .381 |
| NU14 |  |  |  |  |  |  |  |  |  |  |  |  |  | 1.000 | .484 | .435 |
| NU15 |  |  |  |  |  |  |  |  |  |  |  |  |  |  | 1.000 | .689 |
| NU16 |  |  |  |  |  |  |  |  |  |  |  |  |  |  |  | 1.000 |

Table 6: Inter-item correlation matrix in FHC-NU scale

Table 7: Inter-item correlation matrix in FHC-PA scale

| Items | PA1 | PA2 | PA3 | PA4 | PA5 | PA6 | PA7 | PA8 | PA9 | PA10 | PA11 | PA12 | PA13 | PA14 |
| --- | --- | --- | --- | --- | --- | --- | --- | --- | --- | --- | --- | --- | --- | --- |
| PA1 | 1.000 | .589 | .558 | .498 | .388 | .296 | .222 | .235 | .228 | .283 | .284 | .356 | .318 | .302 |
| PA2 |  | 1.000 | .587 | .503 | .432 | .314 | .304 | .259 | .292 | .325 | .247 | .295 | .260 | .263 |
| PA3 |  |  | 1.000 | .566 | .459 | .258 | .328 | .295 | .321 | .323 | .269 | .365 | .262 | .311 |
| PA4 |  |  |  | 1.000 | .508 | .330 | .360 | .348 | .371 | .298 | .251 | .265 | .232 | .228 |
| PA5 |  |  |  |  | 1.000 | .428 | .460 | .368 | .394 | .332 | .213 | .264 | .208 | .303 |
| PA6 |  |  |  |  |  | 1.000 | .603 | .521 | .411 | .397 | .102 | .123 | .121 | .127 |
| PA7 |  |  |  |  |  |  | 1.000 | .625 | .637 | .483 | .129 | .114 | .091 | .195 |
| PA8 |  |  |  |  |  |  |  | 1.000 | .651 | .520 | .157 | .193 | .109 | .205 |
| PA9 |  |  |  |  |  |  |  |  | 1.000 | .556 | .155 | .145 | .103 | .244 |
| PA10 |  |  |  |  |  |  |  |  |  | 1.000 | .227 | .179 | .155 | .132 |
| PA11 |  |  |  |  |  |  |  |  |  |  | 1.000 | .580 | .470 | .347 |
| PA12 |  |  |  |  |  |  |  |  |  |  |  | 1.000 | .708 | .597 |
| PA13 |  |  |  |  |  |  |  |  |  |  |  |  | 1.000 | .585 |
| PA14 |  |  |  |  |  |  |  |  |  |  |  |  |  | 1.000 |
